# Therapeutic hypothermia for cardiac arrest due to non-shockable rhythm: A protocol for systematic review and meta-analysis

**DOI:** 10.1097/MD.0000000000021452

**Published:** 2020-08-28

**Authors:** Yibing Zhu, Huibin Huang, Jingzhi Feng, Yu Ren, Wei Li

**Affiliations:** a Medical Research and Biometrics Center, Fuwai Hospital, National Center for Cardiovascular Diseases, Chinese Academy of Medical Sciences and Peking Union Medical College; b Department of Critical Care Medicine, Beijing Tsinghua Chang Gung Hospital, Beijing, China.

**Keywords:** cardiac arrests, herapeutic hypothermia, meta-analyses, mortality, non-shockable rhythm

## Abstract

**Background:**

The effectiveness of therapeutic hypothermia (TH) for patients following cardiac arrest with non-shockable rhythm is debated. We plan to conduct a systematic review and meta-analysis with all available randomized controlled trials (RCTs) to explore the efficacy and safety of TH in in this population.

**Methods:**

PubMed, EMBASE and Cochrane Library will be searched to identify RCTs published from inception through December 2020 without language restriction. Patients following cardiac arrest due to non-shockable rhythm will be included. The primary outcome is the hospital mortality. The secondary outcome is the favorable neurological outcome. The pooled effects will be analyzed as mean differences using the inverse-variance method for continuous data or as risk ratios using the Mantel–Haenszel method for dichotomous data. Subgroup and sensitivity analyses will be conducted. The Egger's test and/or the funnel plot will be used to test the publication bias. The grades of recommendation assessment, development, and evaluation (GRADE) methodology will be used to assess the quality of evidence. The trial sequential analysis will be used to test whether the meta-analysis is conclusive.

**Results:**

The RCTs on the effectiveness of TH for patients following cardiac arrest with non-shockable rhythm will be systematically reviewed and advance evidence will be provided.

**Conclusion:**

Advanced evidence of TH for cardiac arrest due to non-shockable rhythm will be provided for physicians.

**PROSPERO registration number:**

CRD42020161823.

## 1 Introduction

Therapeutic hypothermia (TH) is recommended for using in adult patients following cardiac arrest with any rhythm.^[1,2]^ Among these patients, cardiac arrest with non-shockable rhythm has a higher mortality.^[3,4]^ However, high evidence of TH supporting its practices remains uncertain in this population; the results of the previous studies showed conflicting results.^[5–11]^ The previous meta-analyses were based mainly on observational studies due to the lack of randomized controlled trials (RCTs) on this issue;^[3,4]^ no meta-analysis on RCTs has been published so far. After that, there have been high quality RCTs published.^[12–14]^ With the updated results, we plan to conduct a systematic review and meta-analysis with all available RCTs to explore the efficacy and safety of TH in in this population.

## 2 Review question

To assess the efficacy and safety of TH for cardiac arrest due to non-shockable rhythm.

## 3 Methods

### 3.1 Study registration

This study was registered on the PROSPERO (registration number: CRD42020161823) in accordance with the PRISMA-P guideline.^[15]^

### 3.2 Search methods

Three electronic databases (PubMed, EMBASE, Cochrane Library) will be searched to identify RCTs published from inception through December 2020 without language restriction. Any potentially relevant reference will also be searched. A search strategy has developed using a combination of “hypothermia OR cooling OR targeted temperature management” and “cardiac arrest OR heart arrest OR ventricular fibrillation” in all fields. We will re-run the search before the final analysis.

### 3.3 Inclusion criteria

#### 3.3.1 Studies

We included only RCTs.

#### 3.3.2 Participants

The study subjects consist of the patients following cardiac arrest due to non-shockable rhythm.

#### 3.3.3 Interventions/comparators

TH (32°C -34°C) as the intervention for cardiac arrest due to non-shockable rhythm, regardless of timing of cooling initiation (in-hospital or pre-hospital). The control groups could be any non-hypothermia management.

#### 3.3.4 Outcomes

The primary outcome is the hospital mortality. The secondary outcome is the favorable neurological outcome (defined as a Cerebral Performance Category score of 1 or 2, or a modified Rankin Scale score of 0 to 2).^[16,17]^

### 3.4 Exclusion criteria

The studies available only in the abstract form will be excluded.

### 3.5 Data collection and analysis

#### 3.5.1 Study screening

The 2 reviewers (ZY and HH) will independently screen the titles and the abstracts of the search results after removal of the duplicates. After the full text obtained, the reference lists will also be screened for potentially relevant studies. The process of selection will be reported as a flow diagram.

#### 3.5.2 Data extraction

The 2 reviewers (ZY and HH) will independently extract the data of the publication information, the characteristics of the studies, subjects, interventions and outcomes using a predesigned form. Any disagreements between the 2 reviewers will be solved in discussion with another 2 reviewers (FJ and RY).

#### 3.5.3 Assessment of study quality

The 2 reviewers (ZY and HH) will independently assess the quality of the RCTs using the Cochrane Collaboration's tool.^[18]^ The grades of recommendation, assessment, development and evaluation (GRADE) methodology will be used to evaluate the quality of evidence.^[19,20]^ Any discrepancies will be discussed with another 2 reviewers (FJ and RY) until an agreement is reached.

#### 3.5.4 Statistical analyses and data synthesis

Review Manager 5.3 will be used to merge data. The synthesis of data requires for at least 3 RCTs. The RCTs meeting the criteria will be summarized in the review whether they are in the qualitive analysis or not. The pooled effects will be analyzed as the risk ratios and 95% confidence intervals for dichotomous data using the Mantel-Haenszel method or the mean differences and 95% confidence intervals for continuous data using the inverse variance method. The significance level of the 2-sided *P* value is .05.

#### 3.5.5 Assessment of heterogeneity

The statistical heterogeneity will be estimated using the *I*^2^ statistic through a chi-square test.^[21]^ The level of statistical heterogeneity (*I*^2^ 0%-40% insignificant, 30%-60% medium, 50%-90% substantial, 76%-100% high) will be evaluated together will methodological and clinical heterogeneity by the 2 reviewers (ZY and HH). A random effect model will be used if there is significant clinical, methodological or statistical heterogeneity. Otherwise, a fixed effect model will be used.

#### 3.5.6 Subgroup and sensitivity analyses

The subgroups of in-hospital TH and pre-hospital TH will be respectively analyzed to optimize the clinical homogeneity in the subsets. The sensitivity analysis will also be performed by excluding each single RCT to test the robustness of the results.

#### 3.5.7 Assessment of publication bias

The Egger test will be used for less than ten RCTs included in the data synthesis. Otherwise, a funnel plot will be chosen.^[22]^

#### 3.5.8 Trial sequential analysis (TSA)

The TSA methodology will be used to adjust the increased risk of errors caused by the data synthesis.^[23]^ The TSA software will be used to analyze the boundary of the sample size and determine whether the present result is conclusive.^[23]^

## 4 Discussion

Since the recommendation for TH for patients following cardiac arrest with non-shockable rhythm remains based on consensus of expert opinion, high quality RCTs and meta-analyses are needed for improvement of the quality of evidence.^[24,25]^ A recent large RCT assessing TH for in-hospital cardiac arrest with non-shockable rhythm observed a higher survival rate with a favorable neurologic outcome in the TH group;^[12]^ the result is similar with some other recent RCTs.^[12,13]^ Our meta-analysis will include the updated high quality RCTs; with the strengths of the largest sample size so far, the rigorous assessment of evidence using the GRADE and TSA methodology, and subgroup and sensitivity analyses, we will provide advanced evidence on TH for patients following cardiac arrest with non-shockable rhythm.

## Author contributions

**Contribution:** Huibin Huang

**Data curation:** Huibin Huang, Yibing Zhu

**Methodology:** Huibin Huang, Yibing Zhu

**Project administration:** Yibing Zhu

**Software:** Jingzhi Feng, Yu Ren

**Supervision:** Wei Li

**Writing – original draft:** Yibing Zhu, Huibin Huang

**Writing – review & editing:** Jingzhi Feng, Yu Ren
